# Contribution of Serological Rapid Diagnostic Tests to the Strategy of Contact Tracing in Households Following SARS-CoV-2 Infection Diagnosis in Children

**DOI:** 10.3389/fped.2021.638502

**Published:** 2021-05-10

**Authors:** Lorelei Charbonnier, Julie Rouprêt-Serzec, Marion Caseris, Marion Danse, Aurélie Cointe, Laure Cohen, Albert Faye, Naïm Ouldali, Jean Gaschignard

**Affiliations:** ^1^Assistance Publique Hopitaux De Paris, Paris, France; ^2^Service de Pédiatrie Générale, Hôpital Robert-Debré, Paris, France; ^3^Service d'Immuno-Hématologie Pédiatrique, Hôpital Robert-Debré, Paris, France; ^4^Service de Neurologie Pédiatrique, Hôpital Robert Debré, Paris, France; ^5^Service de Microbiologie, Hôpital Robert Debré, Paris, France; ^6^INSERM U1137 Infection, Antimicrobiens, Modélisation, Evolution, Paris, France; ^7^Université de Paris, Paris, France; ^8^INSERM U1123 Epidémiologie clinique et évaluation économique appliquées aux populations vulnérables, Paris, France; ^9^Association Clinique et Thérapeutique Infantile du Val de Marne (ACTIV), Saint Maur des Fossées, France

**Keywords:** SARS-CoV-2, contact-tracing, rapid diagnostic test, children, household, RT-PCR-polymerase chain reaction with reverse transcription

## Abstract

**Background:** The contact tracing and isolation of contagious individuals are cornerstones in the control of the COVID-19 pandemic. Strategies to identify household contacts who should be isolated around index children that tested positive for SARS-CoV-2 remain to be clarified. We aimed to compare contact tracing strategies around an index child positive for SARS-CoV-2 using serological rapid diagnostic testing (RDT, chromatography immunoassay).

**Methods:** We conducted a contact tracing study in households of index cases children in the Paris region, France, between May 8 and July 27, 2020. We compared two strategies, one using SARS-CoV-2 reverse transcriptase polymerase chain reaction (RT-PCR) and one combining RT-PCR and serological RDT, initiated once RDT was available. The contacts RT-PCR–/RDT+ were considered to have been previously infected and not requiring quarantine. The primary outcome was the proportion of contacts that could avoid quarantine with the two screening strategies.

**Results:** We included 34 children as index cases. Median age was 7 years. They generated 184 contacts (111 adults, 73 children) tested by RT-PCR: 24/184 (13%) were positive. The strategy combining RDT and RT-PCR was performed in 120/184 contacts (77 adults, 43 children) of 26 index children: 16/120 (13%) were RT-PCR+ and 47/120 (39%) were RDT+. Among the 16 individuals who were RT-PCR+, 14 (87%) were also RDT+. Among the 104 individuals who were RT-PCR–, 33 were RDT+. Hence 33/120 (27%) individuals were not isolated.

**Conclusions:** Following the diagnosis of SARS-CoV-2 infection in children, a strategy combining serological RDT and nasopharyngeal RT-PCR enabled us to identify around one fourth of contacts with past infection and avoid unnecessary quarantine of these individuals.

## Introduction

Since the COVID-19 pandemic started in late 2019, there have been more than 149 million confirmed cases (1), among them only 7.7% are children (2) mostly with mild or asymptomatic presentations (3).

Household contacts are a major source of transmission, with a contamination rate by SARS-CoV-2 estimated between 10 and 16.4% in several studies (4–6), playing a key role in the burden of COVID-19. Thus, identifying infected individuals and quarantining are cornerstones in the control of the COVID-19 pandemic. However, quarantine is not without consequences ranging from social isolation to substantial economic impact (7), especially in families with children. Determining infectious risk for each household with a child tested positive for SARS-CoV-2 is crucial to limit the spread of the virus with appropriate measures for the family.

Contact tracing strategies widely use nasopharyngeal swab for reverse transcriptase polymerase chain reaction (RT-PCR), but this has several limits. A positive RT-PCR might not be systematically associated with a risk of transmission particularly if the cycle threshold (Ct) of detection is high (8). Given that the median incubation period for SARS-CoV-2 is estimated at 5–6 days (9, 10), an early negative RT-PCR does not rule out a latent infection in close contacts. During the early epidemic, self-quarantine and a second RT-PCR between 7 and 14 days after the last contact were thus recommended (11).

Serological rapid diagnostic tests (RDT, chromatography immunoassay) are less invasive and give faster results, but have so far barely been used in contact tracing. Yet, detecting SARS-CoV-2-specific antibodies may contribute to identify a past infection in tested contacts (12). Since our study was conducted during the first wave of the pandemic, we considered the possibility of re-infection in individuals with positive serology highly unlikely.

We hypothesized that combining RDT and nasopharyngeal RT-PCR in household contacts could help to better determine the infectious status of within-households and reconsider the quarantining of family members. Thus, we intended to compare the proportion of contacts that could avoid quarantine with a screening strategy combining RT-PCR and serological RDT compared with the strategy using only RT-PCR.

## Methods

### Study Design

We conducted a prospective cohort study of contact tracing between May 8 and July 27, 2020 in the Paris region, an epicenter of the early COVID-19 epidemic in France.

### Contact Tracing

Index cases were children with a confirmed SARS-CoV-2 infection, whether hospitalized or not, who attended the Pediatric Emergency Department of the Robert Debré University Hospital, Paris, France. A positive RT-PCR was required for inclusion, except for children presenting with pediatric multisystem inflammatory syndrome related to SARS-CoV-2 (MIS-C, including myocarditis, Kawasaki Disease, and atypical KD) who were included provided they had a positive serology. Family members and individuals living in the same place were considered as household contacts.

A contact tracing strategy called COVISAN was set up in the Paris region to break the viral transmission chains, with home visits by nurses to screen close contacts of the patients with SARS-CoV-2 infection. An initial form was filled for every new SARS-CoV-2 infection case to determine the date of symptom onset and collect data on his/her living place and household contacts (identity, age, occupation or attended school, high risk factors, and suggestive symptoms of COVID-19). Protective measures to avoid viral transmission were explained, along with distribution of masks and hydroalcoholic lotions.

### Screening Strategies

Screening with a nasopharyngeal swab for RT-PCR detection of SARS-CoV-2 was offered to all household members. It was performed by nurses or trained medical students. Samples were tested using one of three commercial high-throughput laboratory analyzers [Realstar (Altona)] or quantitative methods [COBAS (Roche) and NeuMODx (Qiagen)], which indicated the Ct for each target in case of a positive result.

From May 8, RDT for a qualitative detection of SARS-CoV-2 IgG and IgM by chromatography immunoassay on whole blood samples from a finger prick became available in our institution. We implemented a new screening strategy combining the nasopharyngeal RT-PCR with a serological RDT: COVID-PRESTO^®^ (specificity 100%, sensitivity 70 and 100%, 11–15 and >15 days after onset of symptoms, respectively, https://www.covid19aaz.com/en/serologic-test/#notice-covid-presto) or NG-TEST^®^/IgG-IgM COVID-19 (specificity 100%, sensitivity 71 and 100%, 11–15 and >15 days after onset of symptoms, respectively, https://ngtest-covid-19.com/en/ng-test-igm-and-igg-all-in-one/). Results were read by professionals, in 10–15 min; RDT was considered positive if positive for IgG and/or IgM, and otherwise negative. In the context of contact tracing, with 13% of individuals positive by PCR, the positive and negative predictive values of our RDT of these tests are estimated at 100 and 87%, respectively.

### Contact Management

Contacts that were tested positive for SARS-CoV-2 RT-PCR were isolated for 14 days, whereas those that tested negative were isolated for 7 days, as recommended during our period of study (11).

For individuals with both nasopharyngeal RT-PCR and RDT performed, we considered those with RT-PCR+, whether RDT+ or –, as potentially contagious individuals and those with RT-PCR–/RDT– as potentially “receptive” of the infection or in incubation phase, all to be isolated. RT-PCR–/RDT+ individuals were considered as having had a prior infection with no need of quarantine.

### Outcome

The primary outcome was the proportion of contacts tested RT-PCR–/RDT+ that could avoid quarantine with the screening strategy combining RT-PCR and serological RDT (sub-group named “Group RT-PCR/RDT”) compared with the strategy using only RT-PCR (“Group RT-PCR”).

### Statistical Analysis

We described patient characteristics as numbers and percentages for categorical variables, and median with interquartile ranges or mean and 95% interval confidence for continuous ones. The Fisher's exact test and the Student's *t*-test were used to compare the proportions of categorical and continuous variables, respectively. A two-sided *p* < 0.05 was considered statistically significant. All statistical analyses were performed using R v3.6.1 (http://www.R-project.org).

### Ethics

This study received approval from the Robert Debré Hospital, Paris, France, institutional review board (decision no. 2020-531).

## Results

### Baseline Characteristics of Eligible Population

A total of 41 patients aged <18 years were identified as index cases between April 17 and July 27, 2020. Median age was 7 years (IQR, 3–13). Among them, 34/41 were RT-PCR positive and 17/41 seropositive for SARS-CoV-2. Twelve children presented with MIS-C (Kawasaki-like syndrome and/or myocarditis): six were RT-PCR– but had a positive serology, four were RT-PCR+/RDT+, and two were RT-PCR+ (RDT not performed). Nine patients presented symptoms suggestive of COVID-19 (fever, respiratory signs, and digestive disorders), and the 25 others were asymptomatic and systematically tested before hospitalization or surgery (Supplementary Table 1). The 41 index cases generated 224 household contacts (138 adults and 86 children), representing a mean of 5.5 contacts per index case.

### Baseline Charactersitics of Population of the Study

**Figure 1:**
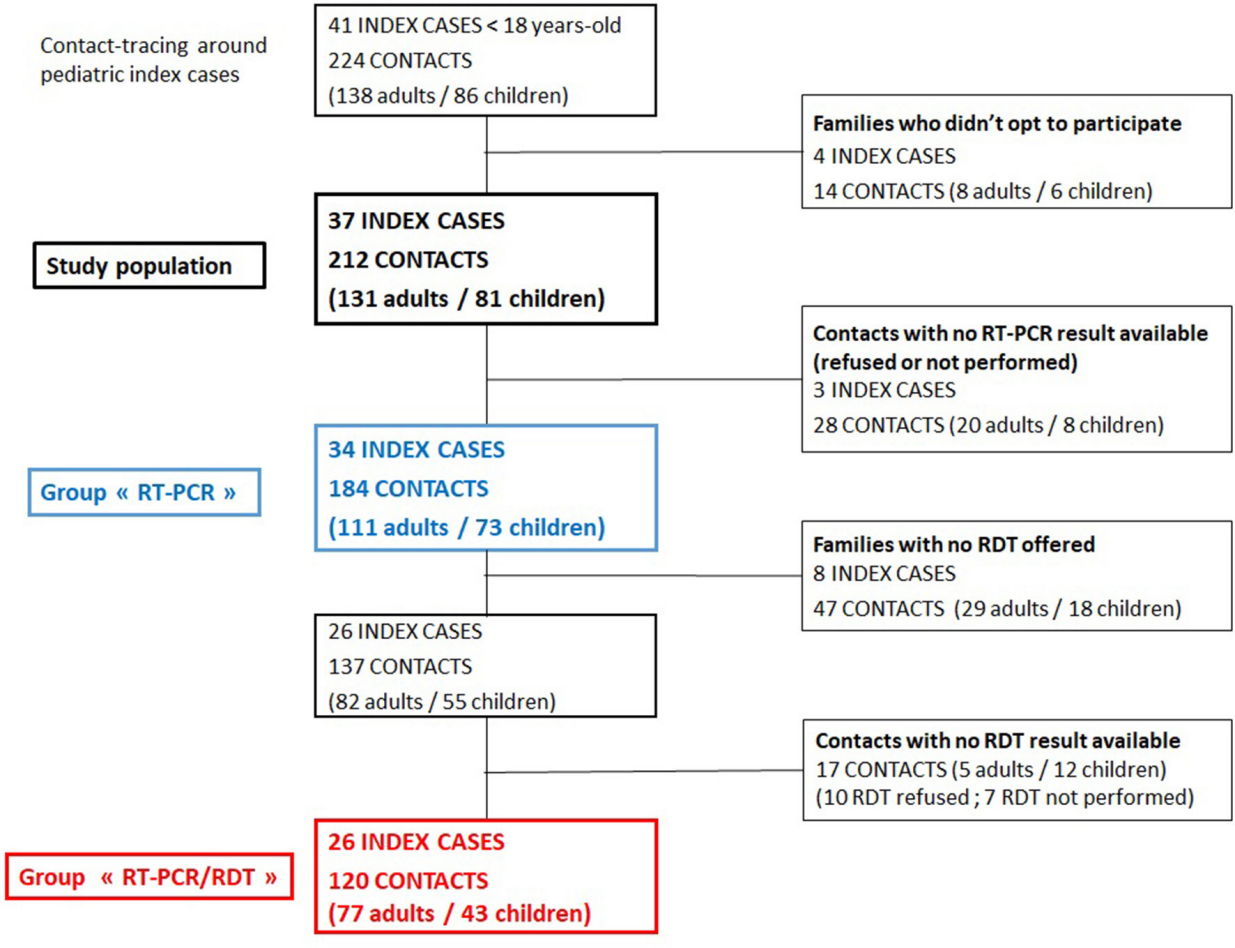
Flowchart of included children cases and household contacts.

Four families (4 index cases, 14 contacts) did not opt to participate in our study and were excluded. RT-PCR results were available for 184 contacts from 34 index cases that were kept for analysis (Figure 1). The demographics and clinical characteristics of these 34 index cases were not different from the 41 eligible ones (Supplementary Table 1).

Among the 184 household contacts (111 adults, 73 children), 47 (47/177, 27%) had a history suggestive of SARS-CoV-2 infection: 31/177 had fever (18%), 19/177 (11%) had respiratory signs, 15/177 (8%) had anosmia, and/or taste loss, 7/177 (4%) had digestive symptoms, and 23/47 (49%) presented signs 4 weeks or more upon testing.

### Comparison of Two Contact Tracing Strategies

We compared the strategy using RT-PCR alone in the group including all 184 contacts (“Group RT-PCR”) to the one using both RT-PCR and serological RDT in the subgroup of 120/184 individuals (77 adults, 43 children) tested both by nasopharyngeal RT-PCR and RDT (“Group RT-PCR/RDT”). The contacts in the two groups had similar clinical presentation (age and symptoms of COVID-19) and the same proportion of positive nasopharyngeal RT-PCR (Table 1). In the “Group RT-PCR,” RT-PCR was positive for 24/184 (13%) contacts (13/111 adults, 11/73 children) (Table 1). In the “Group RT-PCR/RDT,” a total of 47/120 (39%) were RDT+: IgG was positive in 47 cases and IgM in 30 cases. There was, thus, no case of contacts who were IgM+/IgG–. Positivity by RT-PCR was also 13% in this group (9/77 adults, 7/43 children). Among these 16 RT-PCR+ individuals, 14 (88%) were RT-PCR+/RDT+ (7/77 adults and 7/43 children) and 2 (12%) were RT-PCR+/RDT– (2 adults). Among the remaining 104 contacts with a negative RT-PCR, 71 (68%) were RT-PCR–/RDT– (45/77 adults and 26/43 children). Finally, 33/120 (27%) were RT-PCR–/RDT+ (23/77 adults and 10/43 children): these individuals were no longer considered infectious and were not quarantined. Hence, the proportion of contacts to be isolated decreased to 73% (87/120) in the “Group RT-PCR/RDT” vs. 100% (184/184) in the “Group RT-PCR.” The benefit remained unchanged when comparing the “Group RT-PCR/RDT,” (87/120) to the subgroup of contact without RDT (64/64, 100%). The proportion of contacts RT-PCR–/RDT+ around children that did not present with MIS-C was significantly higher than in contacts around children with MIS-C (27/77 vs. 6/43, *p* = 0.02, Supplementary Table 2). The proportion of contacts to be isolated decreased to 65% (50/77) around children without MIS-C, vs. 86% (37/43) around children with MIS-C.

**Table 1:** Household contact characteristics.

	**Group RT-PCR 184 contacts *N* (%)**	**Group RT-PCR/RDT 120 contacts *N* (%)**
Median age (years) [IQR]	23 [13; 42]	24 [15; 42]
**CLINICAL PRESENTATION**		
History suggestive of COVID-19	47/177 (27)	24/118 (20)
Fever	31/177 (18)	17/118 (14)
Anosmia, loss of taste	15/177 (8)	7/118 (6)
RT-PCR performed	184/184	120/120
RT-PCR+	24/184 (13)	16/120 (13)
RT-PCR–	160/184 (87)	104/120 (87)
RDT performed		120/120
RDT+		47/120 (39)
RDT–		73/120 (61)
**ALL CONTACTS**		
RT-PCR+/RDT+		14/120 (12)
RT-PCR+/RDT–		2/120 (2)
RT-PCR–/RDT+		33/120 (27)
RT-PCR–/RDT–		71/120 (59)
**ADULTS**		
**RT-PCR+**	13/111 (12)	9/77 (12)
RT-PCR+/RDT+		7
RT-PCR+/RDT–		2
**RT-PCR**–	98/111 (88)	68/77 (88)
RT-PCR-/RDT+		23
RT-PCR–/RDT–		45
**RDT+**		30/77 (39)
**CHILDREN**		
**RT-PCR+**	11/73 (15)	7/43 (16)
RT-PCR+/RDT+		7
RT-PCR+/RDT–		0
**RT-PCR**–	62/73 (85)	36/43 (84)
RT-PCR–/RDT+		10
RT-PCR–/RDT–		26
**RDT+**		17/43 (40)

## Discussion

We showed that the combined use of serological RDT with RT-PCR in a contact tracing strategy among household contacts of a pediatric SARS-CoV-2 index case enabled us to identify that 27% of household contacts could avoid an unnecessary quarantine, compared with a strategy relying on a sole RT-PCR. To our knowledge, our study is the first to compare these two strategies among household contacts.

Our study was conducted during the first French COVID-19 wave. The usual contact tracing strategy was to identify all contacts and to perform a sole RT-PCR in the 7 days following the contact (11). In this situation, self-quarantining was required for all contacts, 14 days for those tested positive and 7 days for those tested negative. Contacts tested positive for SARS-CoV-2 are now isolated 7 days (13).

SARS-CoV-2 infection can be detected indirectly by evaluating the host immune response to SARS-CoV-2. Specific antibodies against S (spike) and N (nucleocapsid) proteins can be detected from the first week of infection (14). Thus, the detection of specific antibodies in the absence of SARS-CoV-2 RNA should be considered as markers of a prior infection (12, 15). Such individuals, RT-PCR–/RDT+, considered as having been infected but not contagious anymore, could avoid self-quarantine. In our study, 23 adults and 10 children were RT-PCR–/RDT+ and could return to work and school, after only 2 days of isolation awaiting the result of the RT-PCR. Determining infection status of households around SARS-CoV-2-infected children is even more important to relax measures of isolation given the extreme difficulty to isolate children within the household.

All the RDT performed in our study were IgG+, and none were IgM+/IgG–. In our strategy, we thus did not distinguish subgroups according to whether IgG or IgM were positive. However, the probability of being PCR+ is different when the result of the RDT is IgG+ or IgM+/IgG–. Considering that IgM becomes positive sooner than IgG, contacts IgM+/IgG– would be at higher risk of still being contagious than contacts IgG+, but even in the first case, this risk is very low and would have a minor impact on our strategy.

In our study, 16 household contacts of infected children were positive for SARS-CoV-2 by RT-PCR: 14 (88%) also had a positive RDT, while 2 adults (12%) were seronegative. The persistence after several weeks of a positive RT-PCR even in clinically well-individuals raises the question of the infectiousness of positive indidivuals (8, 16, 17). Korea Centers for Disease Control and Prevention investigated 285 persons with “persistently positive RT-PCR” and found no secondary cases among their 790 contacts, and failed to isolate replication-competent SARS-CoV-2 for 108 patients (18); Bullard observed a SARS-CoV-2 Vero cell infectivity only when patients presented symptoms for <8 days and a positive RT-PCR with E gene cycle threshold <24 (8), and Singanayagam showed that the probability of culturing the virus declined to 8% in samples with Ct > 35 and to 6% 10 days after onset of symptoms (19). However, in the context of household contact tracing with large families, one must take into account the possible chains of transmission within the household, including asymptomatic links, making it difficult to rely on the evaluation of the index case.

The presence of specific antibodies is observed in infection evolving for 5 days or more (11) and may correlate with a low infectiousness, even if the RT-PCR is positive: we could thus distinguish individuals RDT+/RT-PCR+ at low risk of transmission from those RDT–/RT-PCR+ as highly contagious. Under these assumptions, we could imagine a contact tracing strategy relying on an initial serological RDT for all identified contacts, and offering nasopharyngeal RT-PCR only to contacts with a negative RDT, reducing substantially the number of RT-PCR performed in a population, the delay before determining the risk of contagiousness for each contact, and the number of contacts requiring quarantine. Considering the persisting burden of SARS-CoV-2 pandemic, this strategy could reduce both the health care resources needed and the economic impact of this pandemic. However, the early PCR test in contacts is not only used to determine the duration of isolation for the contact person but also to identify positive contacts that can, by themselves, generate contacts that should be screened.

Our study focused on limiting the number of people put in quarantine. However, the main goal of contact tracing strategy is to avoid onward transmission. We did not collect information regarding onward transmission with the two strategies in our study, but given the excellent positive predictive value of serological RDT in a population of contacts, the strategy combining serological RDT and RT-PCR was not expected to raise viral transmission by maintaining out-of-quarantine contagious individuals.

The global impact of the strategy combining RDT and RT-PCR relies on the quality of the assay (to correctly classify contacts), the prevalence of the disease (which increases the number of contacts RDT+), but even more on the ability to quickly screen contacts after identifying an index case. The acceptability of the screening strategy is also crucial, as some individuals could refuse the screening for fear of the consequences in terms of isolation. The effect of the strategy we discuss also depends on the setting of the contact tracing. We found that the proportion of contacts that could avoid quarantine was 35% (27/77) around children without MIS-C, vs. 14% (6/43) around children with MIS-C, the former situation being by far the most frequent.

The role children may play in the viral transmission remains unclear, but in literature, children seem to be less contagious than adults (19–21). With our contact tracing strategy, we determined the infectious status of the household members directly after the diagnosis of a SARS-CoV-2 infection in a child: among the 16 adults RT-PCR+, only 2 were RDT– (22, 23).

Our study presents several limitations. First, serological RDT was only available for the contact tracing of 26/34 families, and the intended protocol could only be performed on 120 contacts out of the eligible 224. However, the baseline characteristics of both the index cases and their contacts were similar between these two groups.

Second, we did not confirm RDT results by serologies performed on plasma. In the context of contact tracing, with 13% of individuals positive by PCR, the positive and negative predictive values of the RDT used in our study were estimated at 100 and 87%, respectively, limiting the risk of false positives. The increasing seroprevalence to COVID-19 over time will decrease the risk of false-positive results, if one uses assays with sufficient sensitivity and specificity (95 and 98% required in France, respectively). The risk of a false-positive RDT may depend on the result of the PCR.

Third, during the early epidemics (the setting of our study), contacts with a prior positive PCR were rare, and this possibility was not systematically screened by the nurse in our study. In contacts with a prior positive PCR, RDT has no additional value to diagnose a prior infection. Fourth, the duration of immunization after a first infection with SARS-CoV-2 and its correlation with a positive serology are not fully known. Most individuals with confirmed mild-to-moderate COVID-19 have relatively stable titers of anti-spike antibodies up to 5 months after their infection (24). Meanwhile, cases of reinfection are described, some no later than 2 months after the primary infection (25). Although much remains unknown about immunity after a previous infection, re-infections appear to be rare and mostly occur multiple months after the initial episode. Since our study was conducted during the first wave of the pandemic, we considered the possibility of re-infection in individuals with positive serology highly unlikely.

## Conclusion

The strategy combining RDT and nasopharyngeal RT-PCR among contacts of children positive for SARS-CoV-2 enabled us to detect prior infection in around one fourth of the households and avoid unnecessary quarantine of these individuals. In household contact tracing strategies, combining serological RDT and RT-PCR should be considered.

## Data Availability Statement

The raw data supporting the conclusions of this article will be made available by the authors, without undue reservation.

## Ethics Statement

The studies involving human participants were reviewed and approved by Comité d'Evaluation de l'Ethique des Projets de Recherche de Robert DebréAVIS No 2020-531. Written informed consent from the participants' legal guardian/next of kin was not required to participate in this study in accordance with the national legislation and the institutional requirements.

## Author Contributions

LCh, JG, NO, and AF designed the study. LCh, LCo, JR-S, MD, and JG collected the data. LCh, JG, AF, and NO had a major contribution in the writing of the manuscript. All authors participated in the revison of the manuscript.

## Conflict of Interest

The authors declare that the research was conducted in the absence of any commercial or financial relationships that could be construed as a potential conflict of interest.
