# Supplementary material for: Contribution of Serological Rapid Diagnostic Tests to the Strategy of Contact Tracing in Households Following SARS-CoV-2 Infection Diagnosis in Children
Source: Front Pediatr. 2021 May 10;9:638502. doi: 10.3389/fped.2021.638502 (PMC8141846; doi:10.3389/fped.2021.638502)
Supplement: Supplementary file 1 [file Table_1.docx]

**Supplementary Table 1. Index cases characteristics**

|  | **Eligible children**  **N (%)** | **Group RT-PCR**  **N (%)** | **Group RT-PCR/RDT**  **N (%)** |
| --- | --- | --- | --- |
| Index cases | 41 | 34 | 26 |
| Household contacts | 224 | 184 | 120 |
| Median age (years) [IQR] | 7 [3;13] | 7 [3;12] | 6 [2;9] |
| Age categories (years) |  |  |  |
| 0-5 | 15/41 (37) | 11/34 (32) | 10/26 (38) |
| 5-10 | 12/41 (29) | 12/34 (35) | 10/26 (38) |
| 10-18 | 14/41 (34) | 11/34 (32) | 6/26 (23) |
| 18-60 | 0 | 0 | 0 |
| > 60 | 0 | 0 | 0 |
| **INITIAL PRESENTATION** |  |  |  |
| Fever | 18/41 (44) | 15/34 (44) | 9/26 (35) |
| MIS-C (Kawasaki, myocarditis) | 12/41 (29) | 10/34 (29) | 5/26 (19) |
| COVID-19 suggestive symptoms *(MIS-C excluded)* | 7/41 (17) | 7/34 (21) | 5/26 (19) |
| No symptom of COVID-19 | 22/41 (54) | 17/34 (50) | 16/26 (62) |
| **RESULTS** |  |  |  |
| RT-PCR + | 34/41 (83) | 28/34 (82) | 20/26 (77) |
| SARS-CoV-2 seropositivity *(whether RDT or serology)* | 17/41 (41) | 16/34 (47) | 12/26 (46) |
| RDT performed | 7/41 (17) | 7/34 (21) | 7/26 (27) |
| RDT + | 6/41 (15) | 6/34 (18) | 6/26 (23) |
| Plasmatic serology performed | 11/41 (27) | 10/34 (29) | 6/26 (23) |
| Plasmatic serology + | 11/41 (27) | 10/34 (29) | 6/26 (23) |
