# Supplementary material for: Contribution of Serological Rapid Diagnostic Tests to the Strategy of Contact Tracing in Households Following SARS-CoV-2 Infection Diagnosis in Children
Source: Front Pediatr. 2021 May 10;9:638502. doi: 10.3389/fped.2021.638502 (PMC8141846; doi:10.3389/fped.2021.638502)
Supplement: Supplementary file 2 [file Table_2.docx]

**Supplementary Table 2. Comparison of SARS-CoV-2 results in contacts around index cases with and without MIS-C**

|  | **Index cases without MIS-C** | | **Index cases with MIS-C** |  |  |  |  |  |  |  |
| --- | --- | --- | --- | --- | --- | --- | --- | --- | --- | --- |
|  | **Group RT-PCR**  **110 contacts**  **N (%)** | | **Group RT-PCR/RDT**  **77 contacts**  **N (%)** | **Group RT-PCR**  **74 contacts**  **N (%)** | | **Group RT-PCR/RDT**  **43 contacts**  **N (%)** |  |  |  |  |
| RT-PCR+ | 16/110 (15) | | 12/77 (16) | 8/74 (11) | | 4/43 (9) |  |  |  |  |
| RT-PCR- | 94/110 (85) | | 65/77 (84) | 66/74 (89) | | 39/43 (91) |  |  |  |  |
| RDT+ |  | | 37/77 (48) |  | | 10/43 (23) |  |  |  |  |
| RDT- |  | | 40/77 (52) |  | | 33/43 (77) |  |  |  |  |
| **All contacts** |  | |  |  | |  |  |  |  |  |
| RT-PCR+/RDT+ |  | | 10/77 (13) |  | | 4/43 (9) |  |  |  |  |
| RT-PCR+/RDT- |  | | 2/77 (3) |  | | 0/43 |  |  |  |  |
| **RT-PCR-/RDT+** |  | | **27/77 (35)** |  | | **6/43 (14)** |  |  |  |  |
| RT-PCR-/RDT- |  | | 38/77 (49) |  | | 33/43 (77) |  |  |  |  |
| **Adults** |  | |  |  | |  |  |  |  |  |
| **RT-PCR+** | **7/63 (11)** | | **5/49 (10)** | **6/48 (13)** | | **4/28 (14)** |  |  |  |  |
| RT-PCR+/RDT+ |  | | 3 |  | | 4 |  |  |  |  |
| RT-PCR+/RDT- |  | | 2 |  | | 0 |  |  |  |  |
| **RT-PCR-** | **56/63 (89)** | | **44/49 (90)** | **42/48 (87)** | | **24/28 (86)** |  |  |  |  |
| RT-PCR-/RDT+ |  | | 19 |  | | 4 |  |  |  |  |
| RT-PCR-/RDT- |  | | 25 |  | | 20 |  |  |  |  |
| **RDT+** |  | | **22/49 (45)** |  | | **8/28 (29)** |  |  |  |  |
| **Children** |  | |  |  | |  |  |  |  |  |
| **RT-PCR+** | **9/47 (19)** | | **7/28 (25)** | **2/26 (8)** | | **0/15** |  |  |  |  |
| RT-PCR+/RDT+ |  | | 7 |  | | 0 |  |  |  |  |
| RT-PCR+/RDT- |  | | 0 |  | | 0 |  |  |  |  |
| **RT-PCR-** | **38/47 (81)** | | **21/28 (75)** | **24/26 (92)** | | **15/15** |  |  |  |  |
| RT-PCR-/RDT+ |  | | 8 |  | | 2 |  |  |  |  |
| RT-PCR-/RDT- |  | | 13 |  | | 13 |  |  |  |  |
| **RDT+** | |  | **15/28 (54)** | |  | | | **2/15 (13)** |  |  |
